# Synergistic effects of mannoprotein and ultrasound on the interfacial properties, flavor, and structure of yeast protein

**DOI:** 10.1016/j.ultsonch.2025.107372

**Published:** 2025-05-02

**Authors:** Jin Luo, Li Liang, Yongzhao Bi, Xialei Liu, Kaina Qiao, Zunying Liu, Xiangzhao Mao, Baoguo Sun, Yuyu Zhang

**Affiliations:** a Key Laboratory of Geriatric Nutrition and Health (Beijing Technology and Business University), Ministry of Education, Beijing 100048, China; b Food Laboratory of Zhongyuan, Beijing Technology and Business University, Beijing 100048, China; c Key Laboratory of Flavor Science of China General Chamber of Commerce, Beijing Technology and Business University, 100048, China; d College of Food Science and Engineering, Ocean University of China, Qingdao 266100, China

**Keywords:** Yeast protein, Mannoprotein, Ultrasound, Structure, Interfacial properties, Flavor enhancement

## Abstract

To expand the application of yeast protein (YP) in the food industry, the synergistic effects of mannoprotein (MP) and ultrasound (US) treatment were explored to improve its solubility, flavor, and structural properties. Results showed that YP and MP formed YP-MP conjugation in 1:3 (*w*:*w*), after reacting with citric acid and sodium bicarbonate for 4 h at room temperature and ultrasonic treatment at 1000 W for 30 min. The YP-MP-US sample achieved a maximum protein yield of 54.82 ± 1.67 %. Furthermore, the protein particle size was reduced from 257.67 nm to 159.33 nm after the treatment, thereby improving its solubility, emulsifying, and foaming capacities. Sensory evaluations, combined with E-tongue, E-nose, and HS-SPME-GC–MS analyses, revealed a significant reduction in the unpleasant yeast flavor of YP (*p* < 0.05). Additionally, the synergistic treatment had altered the conformation and structure of YP, which were confirmed by the notable increase in β-sheet, free sulfhydryl (–SH) groups, surface hydrophobicity, and intrinsic fluorescence while promoting a looser and finer aggregation microstructure. In conclusion, the synergistic treatment significantly improved protein solubility and flavor, thereby enhancing its processing functionality.

## 1 Introduction

In recent years, the demand for protein in both per capita and global consumption has been increasing annually, driving the exploration of sustainable protein alternatives due to the insufficient supply of conventional proteins [1]. Yeast protein (YP), a novel microbial protein, exhibits a nutritional value comparable to that of whey protein and surpasses traditional plant protein in terms of hypoallergenic properties, heat stability, acid and alkaline resistance, and versatile application characteristics [2]. Furthermore, the production of yeast protein is sustainable and environment-friendly, as it is not restricted by agricultural practices or environmental conditions [3,4]. Yeast protein shows excellent nutritional and functional properties for the food processing industry, including high protein content and superior water-holding capacity, making it suitable for direct consumption or further processing [5]. However, preliminary studies have identified low solubility and a pronounced yeast off-flavor as major limitations to a broader application of YP in food products [6]. Therefore, modifying yeast protein is essential to improve its usability in the food industry.

Polysaccharides could form complexes with protein molecules to forms complex, enhancing the physicochemical properties of proteins such as dispersibility and emulsification [[7], [8], [9]]. The efficacy of polysaccharides is often constrained by factors involving molecular structure, pH, ionic strength, temperature, and biocompatibility. Moreover, the addition of polysaccharides alone may selectively bind to only specific structural domains of proteins, thereby promoting protein aggregation and reducing their dispersion and solubility in solution [10]. Ultrasound (US) is an environmentally friendly and cost-effective non-thermal modification technique with significant potential in food applications [11]. US treatment is known to mitigate off-flavors by enhancing the binding capacity between flavor compounds and proteins through cavitation effects, as demonstrated in duck liver protein, soybean milk, and pea protein [[12], [13], [14]]. Additionally, the ultrasonic processing induces acoustic cavitation, exposing hydrophobic groups of protein and reducing particle size. Studies have shown that ultrasonic can enhance the physicochemical and functional properties of proteins, including solubility, emulsification, and foaming abilities [15,16]. Nevertheless, the effects of ultrasonication alone on protein functionality are limited [17]. Notably, some studies have been conducted on ultrasound to prepare protein-polysaccharide complexes with enhanced physicochemical properties [[18], [19], [20]]. Therefore, we considered polysaccharide and synergistic US treatment for protein modification. MP, a key component of yeast cell walls, is composed of hydrophilic mannose polymers covalently linked to a protein backbone. MP exhibits significant foaming and foam stabilization properties, as well as health-promoting effects [[21], [22], [23]]. As a natural functional ingredient, MP is easily accepted by consumers.

In this study, the synergistic effects of MP and US treatment on enhancing the solubility and flavor of YP were investigated by analyzing its functional and structural properties. The findings aim to provide a theoretical foundation for improving YP characteristics and expanding its applications in the food industry. By potentially replacing plant and animal proteins, the adoption of YP could reduce dependence on traditional livestock and agricultural systems.

## 2 Materials and methods

### 2.1 Materials

YP and MP were supplied by Angel Yeast Co., Ltd. (Yichang, Hubei, China). Citric acid was sourced from Beijing Taste Food Source Food Technology Co., Ltd. (Beijing, China), while sodium bicarbonate was obtained from Yonghui Supermarket Co., Ltd. (Fuzhou, Fujian, China); all materials are food grade.

### 2.2 Preparation of YP sample

To enhance the dissolution of the YP, the solution containing 0.1 g/mL YP was prepared by thoroughly dissolving citric acid in the ratio of 1:3 (*w*:*w*) with yeast protein. This mixture was then allowed to react for 3 h at room temperature. Afterward, an equal amount of sodium bicarbonate was added in the same ratio, and the reaction was allowed to proceed for 1 h under the same conditions. Upon completion of the reaction, the solution was centrifuged at 4 °C for 5 min at 8000 rpm, and this step was repeated three times. The resulting sample was called YP-BM.

YP-US sample: YP-BM solution (200 mL) was subjected to US treatment using an ultrasonic cell disruptor (SCIENTZ-IID, Ningbo Scientz Biotechnology Co., Ltd, Ningbo, China) at 1000 W for 30 min. The solution was then centrifuged three times once the treatment was completed.

YP-US-MP sample: MP was added to the YP-US solution at a mass ratio of 1:10 (*w*:*w*) of YP to ensure complete dissolution, followed by centrifugation.

YP-MP sample: MP was added to the YP solution at a mass ratio of 1:10 (*w*:*w*), followed by the same procedure used to prepare the YP-BM sample.

YP-MP-US sample: Perform the same ultrasound and centrifugation procedure on the obtained YP-MP solution as in the preparation of the YP-US sample.

These YP products of each group were obtained when the above protein mixtures were processed with distilled water completely dissolved in a ratio of 1:5 (*m*:*v*) through a spray dryer (B-29, Büchi AG, Zurich, Switzerland) at the inlet temperature of 140 ℃, outlet temperature of 83 ℃, aspirator of 100 %, and pump of 7 %.

### 2.3 Characterization of yeast protein interfacial properties

#### 2.3.1 Particle size and ζ-potential

The particle size and ζ-potential of the samples (1 mg/mL) were conducted using the Zeta Sizer Nano ZS90 (Malvern Instruments, Ltd., U.K.), following the modified method of Yang et al. [24].

#### 2.3.2 Solubility

Solubility measurements were conducted following the method outlined by Yang et al. [25]. These samples (1.0 mg/mL) were adjusted to pH 7.0 using 0.1 M HCl and NaOH. Then, centrifugated (9,460 rpm for 15 min at 4 ℃) and collected the supernatant, the protein concentration was determined using a BCA protein assay kit (Thermo Fisher, Waltham, MA, USA).

#### 2.3.3 Emulsification properties

The emulsifying activity (EAI) and emulsifying stability (ESI) of the proteins were evaluated according to the methods of Wang et al. [26]. 15 mL sample (1.0 %) was homogenized (T25, IKA-Werke GmbH & CO. KG, Germany) with 5 mL of soybean oil at 12,000 rpm for 1 min. At 0 and 10 min, 50 μL of the emulsion was collected and mixed with 5 mL of 0.1 % SDS. The absorbance was determined at 500 nm, with 0.1 % SDS serving as the blank.

(1) $$\mathrm{EAI}\left(m^{2}/g\right)=\frac{2\times 2.303\times A_{0}\times N}{C\times \varphi \times 1000}$$

(2) $$ESI\left(min\right)=\frac{A_{0}}{A_{0}-A_{10}}\times 10$$

where N is the dilution factor (100), C is the protein concentration before emulsion formation (g/mL), and φ is the volume fraction of oil in the emulsion (0.25).

#### 2.3.4 Foaming properties

The foaming properties of proteins were assessed using the method described by Zhao et al. [27]. 5 mL sample was homogenized at 15,000 rpm for 1 min. After agitation, the foam volume at 0 min (V_0_) was measured and compared to the original liquid volume (V_1_) to evaluate the foaming ability (FA). The foaming stability (FS) was determined by comparing foam volume at 10 min (V_2_) and V_1_. Equations (3), (4) were utilized to calculate FA and FS:

(3) $$FA\;\left(\%\right)=\frac{V_{1}}{V_{0}}\times 100\%$$

(4) $$FS\;\left(\%\right)=\frac{V_{2}}{V_{1}}\times 100\%$$

### 2.4 Evaluation of yeast protein flavors

#### 2.4.1 Sensory evaluation

A total of 12 staff members (six males and six females, aged 20–30) were recruited from Beijing Technology and Business University, all of whom had relevant experience and systematic training in sensory analysis. Participants used paper ballots featuring rating scales and a scoring system from 0 to 5 points to assess flavor intensity (0–3 for general aroma and yeast flavor; 3–5 for harmonious and abundant aroma without off-flavors) and taste (0–3 for average taste with graininess; 3–5 for mellow taste with no noticeable graininess). These samples were evaluated in triplicate. All panelists provided informed consent and volunteered to participate in this study. The Ethics Committee of Beijing Technology and Business University approved all procedures related to the sensory evaluation (Beijing, China, No. 2023050).

#### 2.4.2 E-nose

The E-nose analysis was conducted following the method described by Weng et al. [28]. 4 mL sample of 10 % YP solution was placed in a 20 mL glass vial and heated for 1 h at 45 °C. Initially, the instrument was cleaned to ensure a stable aroma profile before sample analysis began. Samples were analyzed duration of 60 s at an injection flow rate of 400 mL/min.

#### 2.4.3 E-tongue

Taste analysis was conducted using an E-tongue (TS-5000Z, Insent Inc., Atsugi-Shi, Japan) following the methodology outlined by Zhu et al. [29]. In brief, 10 g of spray-dried samples were mixed with 100 mL of deionized water in 250 mL beakers. The mixture was stirred, and filtered through two layers of filter paper. The resulting filtrate was then analyzed using the E-tongue. Before measurement, the E-tongue probe was preheated, calibrated, and its electrodes activated to ensure the accuracy of the collected data.

#### 2.4.4 HS-SPME-GC–MS

4.0 mL YP samples were added to a 20 mL headspace vial, sealed with a polytetrafluoroethylene-silicone septum, and incubated at 45 °C for 20 min. Volatile compounds were extracted using a 65 µm DVB/PDMS fiber needle (Supelco, Bellefonte, PA) for 40 min at 45 °C, and desorbed at 250 °C for 5 min. Analyses were conducted on an Agilent 7890B GC system with a quadrupole mass filter (Agilent Technologies, Santa Clara, CA), separating volatile compounds on a DB-WAX capillary column (30 m × 0.25 mm × 0.25 μm). The temperature increase program began at 35 °C (1 min held), increased to 100 °C at 4 °C/min (1 min held), then to 170 °C at 2 °C/min (1 min held) and finally to 220 °C at 5 °C/min (5 min held).

### 2.5 Structural characterization of yeast proteins

#### 2.5.1 Fourier transform infrared spectroscopy (FTIR)

The FTIR analysis was performed using a modified method from Cheng et al. [30]. 1 mg of dried YP samples was mixed with 100 mg of KBr, thoroughly ground, and then pressed into a transparent sheet with a pressure of 5 × 10^7^ Pa. The samples were placed into an infrared spectrometer (Nicolet iS50, Thermo Fisher Scientific Inc., USA) for 64 scans in the band of 500–4000 cm^−1^.

#### 2.5.2 Intrinsic fluorescence spectroscopy

The measurements were conducted following the method of Guo et al. [31]. The samples (0.2 mg/mL in PBS) were evaluated using a fluorescence spectrometer (F-7000, Hitachi, Japan). The excitation wavelength is 280 nm, the scanning range ranges from 290 nm to 450 nm, excitation slit and emission slit at 2.5 nm.

#### 2.5.3 Surface hydrophobicity (H0)

Surface hydrophobicity was assessed with the modified method of Chen et al. [32]. 20 μL solution of 8 mM ANS (1-anilino-8-naphthalenesulfonate) was added to 4 mL of YP samples (1 mg/mL), mixed thoroughly, then reacted in the dark for 15 min. Fluorescence intensity was measured with a Multi-function Enzyme Labeler (M200 Pro, Tecan Group Ltd., Männedorf, Switzerland), with an excitation wavelength of 390 nm and emission collected at 470 nm.

#### 2.5.4 Free sulfhydryl group (–SH) and disulfide bond (S–S) contents

Sulfhydryl group (–SH) and disulfide bond (S–S) contents were analyzed with a modified method from Feng et al. [33]. For the determination of free –SH content, YP solution (1 mL, 5 mg/mL) was mixed with 5 mL of Tris-Glycine buffer (1.04 g Tris, 0.69 g Glycine, 0.12 g EDTA, distilled water fixed at 100 mL) was mixed with 20 μL of Ellman (0.04 g DTNB, Tris-Glycine buffer solution fixed at 10 mL The reagent was mixed with 20 μL of Ellman (0.04 g DTNB, Tris-Glycine buffer solution, 10 mL), and the absorbance value was read at 412 nm after standing for 15 min. For the determination of total sulfhydryl content, 0.5 mL of 10 % YP solution, 5 mL of 8 mol/L urea buffer (10.4 g Tris, 6.9 g Glycine, 1.2 g EDTA, 480 g urea, 1000 mL of distilled water, pH 8.0) were mixed with 20 μL of Ellman's reagent, and the absorbance values were read under 412 nm after 15 min of standing. The formulae for calculating the content of sulfhydryl groups and disulfide bonds are shown in (5) and (6):

(5) $$-SH\;\left(\mu M/g\right)=\frac{75.53\times A_{412}\times D}{C}$$

(6) $$S-S\;\left(\mu M/g\right)=\frac{\mathrm{Total}\;-SH-Free\;-SH}{C2}$$

where A_412_ denotes the absorbance at 412 nm; C denotes the sample concentration (mg/mL); and D denotes the dilution factor.

#### 2.5.5 SEM

The morphology of the spray-dried samples was observed using SEM at an accelerating voltage of 5 kV and magnifications of 200×, 1000×, 5000×, and 10,000 × . Before imaging, the dried samples were coated with gold using an ion sputter coater.

### 2.6 Statistical analysis

All experiments were repeated in triplicate, with results expressed as mean ± standard deviation. Significance was assessed using ANOVA with SPSS 26.0 software, where *p* < 0.05 was deemed significant. Graphs and further analysis were conducted using Origin 2021 software.

## 3 Result and discussion

### 3.1 Characterization of yeast proteins interfacial properties

#### 3.1.1 Particle size and ζ-potential

**Fig. 1:**
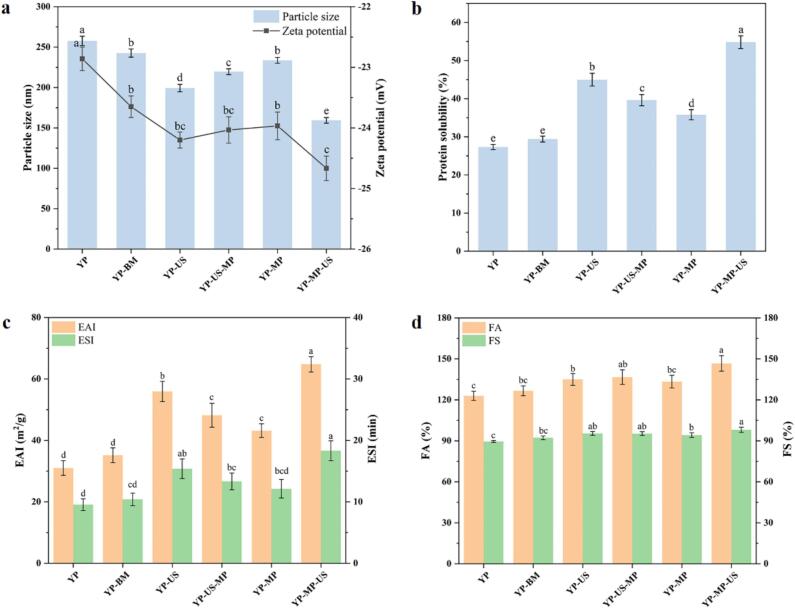
Effect of synergistic treatment of MP and US on YP interfacial properties. Particle size and Zate potential (a), Solubility (b), Emulsification properties (c), and Foaming properties (d).

Protein aggregation is characterized by particle size and ζ-potential, which reflect molecular protein–protein interactions [34]. Compared to the YP material, five different YP samples displayed a decline in particle size, which suggested that five different treatments have some effect on improving the YP (Fig. 1a). Notably, the particle size of the synergistic US treatment sample of YP-US and YP-MP-US was significantly reduced (*p* < 0.05) compared to the YP-BM and YP-MP, meanwhile, the particle size of the YP-US samples was significantly reduced as compared to the YP-MP samples, which suggests that US has a significant effect on improving the degree of yeast protein aggregation. Interestingly, we found a significantly decreased particle size in YP-MP-US samples compared to YP-US-MP, which may be attributed to the fact that the MP improves stability and dispersion through reduced interaction forces, and synergistic US effect on protein particle size distribution is more homogeneous, maintains better stability, and reduces precipitation and aggregation phenomena. In this case, we observed that the protein particle size decreased as the zeta potential was increased. This might be attributed to the fact that smaller particles have a larger surface area and more charge regions, which contributed to the enhancement of inter-particle repulsion, thus increasing the stability of the potential. Overall, the YP-MP-US sample exhibited a significant decrease in average particle size (159.33 nm) and an increase in absolute ζ-potential (−24.66 mV) compared to other groups, suggesting that the synergistic MP and US treatment significantly impact the surface charge distribution of YP, enhancing the stability of the system.

#### 3.1.2 Solubility

Solubility is crucial for proteins to serve effectively as functional components in food, as they need to have high solubility to act as emulsifiers and foaming agents [32]. As shown in Fig. 1b, the YP-MP-US sample exhibited the highest solubility value (54.82 ± 1.67 %). Notably, US treatment has a significant effect on the solubility of YP samples, with marked differences observed between YP-BM and YP-US, YP-MP and YP-MP-US, and YP-MP and YP-US samples. Cavitation-induced shear stress can break hydrogen bonds and disrupt hydrophobic, electrostatic, and other non-covalent interactions in proteins, causing conformational changes that convert proteins from an insoluble to a soluble state [35]. Meanwhile, the higher solubility of YP-MP-US compared to YP-US-MP was attributed to the fact that YP-MP conjugates had a higher solubility than YP itself when the simultaneous addition of MP, and appropriate synergistic US treatment can loosen the molecular structure of YP, further enhancing the protein-water interactions [35]. It was consistent with the results of particle size (Fig. 1a), and smaller particle sizes improved the protein-water interaction [36]. Furthermore, studies indicate that a larger absolute ζ-potential boosts electrostatic repulsion between particles (Fig. 1a), which can modify protein aggregation and enhance protein solubility [37].

#### 3.1.3 Emulsification properties analysis

EAI measures the protein's ability to adsorb onto the surfaces of oil droplets, while ESI assesses the protein's capacity to form stable emulsions that resist phase separation [16]. As illustrated in Fig. 1c, the EAI values of each sample in the order of YP-MP-US > YP-US > YP-US-MP > YP-MP > YP-BM > YP, it indicated that the synergistic MP and ultrasonic treatment could improve the EAI properties of YP. Meanwhile, the highest ESI value (18.31 min) of YP-MP-US. This suggested that synergistic treatment of MP and US had a significant effect on the ability to improve the emulsification and emulsion stability of YP (*p* < 0.05). Meanwhile, Malik et al. [16] reported that protein solubility and H0 were critical for emulsification, which is consistent with the results that synergistic treatment of MP and US could significantly improve the solubility of YP.

#### 3.1.4 Foaming properties analysis

The appearance and morphological stability of processed foods depend on foaming properties [38]. As shown in Fig. 1d, YP exhibited the lowest FA of 123.33 ± 3.33 %, and YP-MP-US had the highest FA of 146.67 ± 5.67 %. The FA values for the individually treated samples with ultrasound (YP-US) and mannoprotein (YP-MP) were 135 ± 4.33 % and 133.33 ± 4.67 %, respectively. Compared to YP-BM and YP-MP, YP-US and YP-MP-US exhibited higher FA values. This improvement can be attributed to the US treatment, which enhanced solubility, reduced particle size, and facilitated adsorption at the air–water interface, thereby lowering the interfacial energy barrier and improving FA. [39,40]. In addition, YP-MP-US exhibited the highest FS value of 98.10 ± 1.88 %. The FS of the protein is primarily associated with the interfacial network formed by its adsorption at the air–water interface, which suggested synergistic treatment of MP and US provide the most effective protection against bubble coalescence and disproportionation, thereby enhancing foam stability.

### 3.2 Evaluation of yeast protein flavor properties

#### 3.2.1 Sensory evaluation

**Fig. 2:**
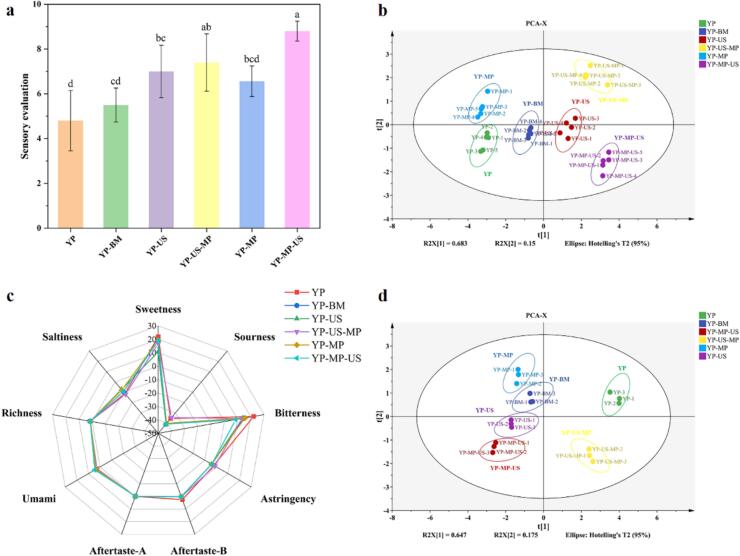
Effect of synergistic treatment of MP and US on improving the flavor perception of YP. Sensory evaluation (a), E-nose (b), E-tongue (c), and the PCA plot (d).

As illustrated in Fig. 2a, the sensory evaluation of the yeast protein samples was improved for all five different treatments compared to the YP material, in which the YP-MP-US sample had the highest score of 8.80 ± 0.45. Compared with YP-BM and YP-MP, the scores of YP-US and YP-MP-US samples synergistic US treatment were significantly improved (*p* < 0.05), which suggested that US treatment can improve the overall sensory properties of yeast proteins. Meanwhile, compared with YP-BM and YP-US, the scores of YP-MP and YP-US-MP samples were improved, which indicated that the addition of MP to the treatment process could improve the sensory properties of YP. In addition, the YP-MP-US samples scored significantly higher than the YP-US-MP samples (*p* < 0.05), which indicated that the addition of MP before YP modification was more effective. In conclusion, this study demonstrated that synergistic MP and US treatment could moderately improve the sensory properties of YP. Therefore, further investigation was carried out by E-nose, E-tongue, and GC–MS.

#### 3.2.2 E-nose analysis

We conducted PCA analysis on the E-nose data, which revealed a total cumulative variance contribution of 83.3 % for PC1 and PC2 (Fig. 2b). Therefore, PC1 and PC2 effectively capture the key characteristics of the YP samples. The variance contribution of PC1 was greater than that of PC2, indicating that sample variation increases with the distance along the PC1 axis, we observed that the left half-axis mainly clustered with YP, YP-MP, and YP-BM samples, and the right half-axis clustered with YP-US-MP, YP-US, and YP-MP-US samples, which distinguished all the samples well according to whether they were US-treated at all. Additionally, the YP-US-MP, YP-US, and YP-MP-US samples were positioned farther from the control group on the PC2 axis, with no overlapping regions, indicating that a significant difference in flavor characteristics due to synergistic MP and US treatment, aligning with the sensory evaluation results.

#### 3.2.3 E-tongue analysis

As shown in Fig. 2c, we found that the intensities of sweetness and bitterness in the differently treated YP samples were lower than those of the YP material, especially the bitterness intensity. Interestingly, compared with YP-BM and YP-MP, the YP-US and YP-MP-US samples significantly reduced the bitter intensity, which could be attributed to the ability of ultrasound to disrupt the protein structure, thereby increasing the release of amino acids. Meanwhile, the taste properties of YP-MP-US samples were better than YP-US-MP, which might be due to the conjugation of MP with YP to form YP-MP conjugates and the bitter taste was significantly reduced after the synergistic US treatment (*p* < 0.05), which might be related to the change of protein structure.

To gain further insights into the E-tongue data, PCA was performed to emphasize the differences in the distribution of nonvolatile compounds. The PCA results for the nonvolatile compounds in the various treated YP samples were illustrated in Fig. 2d. The cumulative variance of 82.20 % (PC1 and PC2), indicating that these two principal components captured the key information on taste. According to the PCA plot, the YP material alone resides in the first quadrant, demonstrating that different treatments improved the taste properties of YP. At the same time, the non-US YP samples are all positioned in the positive half-axis of the Y-axis, while the US-treated YP samples are all situated in the lower half-axis, clearly distinguishing the effects of ultrasound treatment. Meanwhile, the YP-US-MP samples were located in the fourth quadrant alone, while the YP-US and YP-MP-US samples were positioned in the third quadrant. Thus, the addition of MP after US treatment may impart stronger taste properties of MP itself to the samples, which is consistent with the results of the sensory evaluation.

#### 3.2.4 HS-SPME-GC–MS analysis

**Fig. 3:**
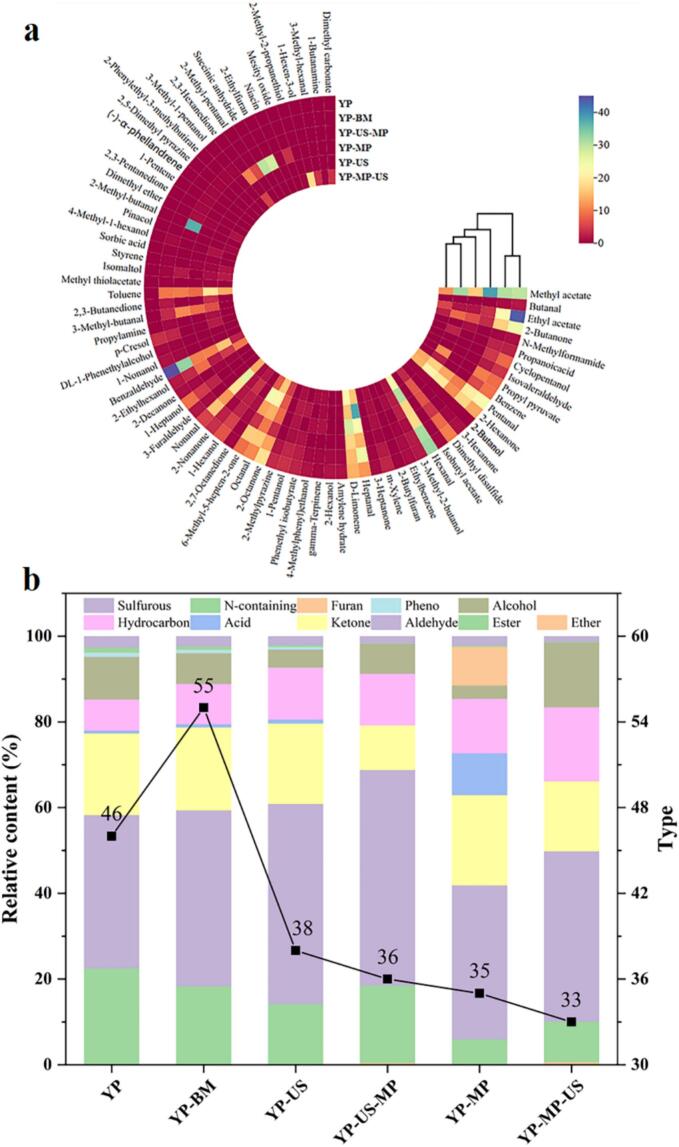
Effect of synergistic treatment of MP and US on improving the volatile flavor of YP. Flavor heatmap (a), Relative content, and type (b).

The distinct yeast flavor, a key factor influencing consumer acceptance of yeast protein products, significantly restricts their applications in the food industry [41]. These undesirable flavor-causing volatiles are affected by a multitude of factors, such as metabolic pathways and environmental factors, etc. To further assess the volatile components in yeast protein under various conditions involving the synergistic treatment of MP and US, HS-SPME-GC–MS was employed to monitor these volatile compounds. Fig. 3a presented the relative content of these volatile compounds under different conditions. Among these, we detected 76 volatile compounds identified in six samples, including 8 esters, 13 aldehydes, 14 ketones, 4 acids, 9 hydrocarbons, 18 alcohols, 1 phenol, 5 N-containing compounds, 2 furans, 1 ether, and 1 sulfurous compound. Among these, the concentration of aldehydes in YP raw material was the highest (35.30 %), followed by that of esters (22.48 %) and ketones (19.08 %), while aldehydes also dominated in the other YP samples, the type of aldehyde species was reduced (Fig. 3b). In terms of individual compounds, 18 volatile compounds were detected in these YP samples, including 3-methyl-2-butanol, ethyl acetate, pentanal, 2-hexanone, dimethyl disulfide, hexanal, m-Xylene, heptanal, D-limonene, 2-octanone, octanal, 6-methyl-5-hepten-2-one, 1-hexanol, 2-nonanone, nonanal, 2-ethylhexanol, and benzaldehyde (Table. 1).

**Table 1:** Identification and quantification of aroma-active compounds in yeast proteins under different treatments by HS-SPME-GC–MS.

Flavor compound	CAS	Concentration (µg/kg)						Flavor description
		YP	YP-NUS	YP-US	YP-US-MP	MP-YP-NUS	MP-YP-US	
3-methyl-2-butanol	598–75-4	1.81 ± 0.53^ab^	1.90 ± 0.33^ab^	1.99 ± 0.47^ab^	0.96 ± 0.33^b^	2.43 ± 0.47^a^	1.19 ± 0.07^b^	fruity
ethyl acetate	141–78-6	43.89 ± 5.14^a^	22.72 ± 1.98^b^	1.54 ± 0.37^c^	3.64 ± 1.37^c^	1.57 ± 0.63^c^	5.48 ± 0.57^c^	pineapple
pentanal	110–62-3	23.41 ± 0.67^a^	20.20 ± 1.24^ab^	16.99 ± 0.07^bc^	9.89 ± 0.87^d^	13.24 ± 3.67^cd^	13.42 ± 0.73^cd^	almond, malt, pungent
2-hexanone	591–78-6	13.25 ± 0.38^bc^	17.36 ± 0.97^ab^	21.46 ± 4.16^a^	11.36 ± 0.43^c^	19.75 ± 1.33^a^	14.20 ± 0.67^bc^	ether
dimethyl disulfide	624–92-0	10.20 ± 0.82^a^	8.64 ± 0.28^a^	7.08 ± 0.47^ab^	4.10 ± 0.27^bc^	6.70 ± 3.23^ab^	3.08 ± 0.97^c^	onion, cabbage, putrid
hexanal	66–25-1	32.30 ± 3.34^a^	31.53 ± 1.47^a^	30.77 ± 1.69^a^	18.01 ± 2.33^c^	17.43 ± 0.37^c^	24.08 ± 0.77^b^	grass, tallow, fat
m-Xylene	108–38-3	2.81 ± 0.02^a^	1.93 ± 0.67^ab^	1.04 ± 0.21^bc^	0.45 ± 0.03^c^	1.14 ± 0.83^bc^	0.88 ± 0.03^bc^	plastic
heptanal	111–71-7	12.07 ± 0.37^c^	25.09 ± 1.53^b^	38.12 ± 3.47^a^	14.25 ± 1.27^c^	22.44 ± 3.86^b^	15.92 ± 0.87^c^	fat, citrus, rancid
D-limonene	138–86-3	14.01 ± 1.14^c^	14.90 ± 0.97^c^	15.80 ± 0.97^c^	17.31 ± 1.37^c^	28.14 ± 3.63^a^	22.01 ± 0.77^b^	lemon, orange
2-octanone	111–13-7	18.67 ± 0.83^a^	12.85 ± 0.51^b^	7.03 ± 0.24^cd^	3.29 ± 0.37^e^	8.77 ± 3.43^c^	4.88 ± 0.27^de^	soap, gasoline
octanal	124–13-0	11.08 ± 0.32^cd^	16.28 ± 0.46^b^	21.48 ± 1.79^a^	9.84 ± 1.27^d^	14.01 ± 2.67^bc^	15.91 ± 1.67^b^	fat, soap, lemon, green
6-methyl-5-hepten-2-one	110–93-0	9.00 ± 0.23^d^	16.49 ± 1.43^b^	24.03 ± 0.37^a^	3.80 ± 0.27^e^	12.98 ± 3.27^c^	7.14 ± 0.73^de^	citrus, fruity
1-hexanol	111–27-3	6.68 ± 0.24^a^	5.25 ± 0.53^b^	3.82 ± 0.47^c^	2.68 ± 0.13^d^	2.39 ± 0.57^d^	1.82 ± 0.23^d^	resin, flower, green
2-nonanone	821–55-6	3.63 ± 0.23^a^	2.48 ± 0.14^b^	1.33 ± 0.15^c^	0.56 ± 0.07^d^	1.66 ± 0.16^c^	0.80 ± 0.13^d^	hot milk, soap, green
nonanal	124–19-6	5.11 ± 0.71^e^	15.87 ± 0.51^c^	26.62 ± 0.47^a^	12.19 ± 0.33^d^	19.02 ± 2.37^b^	13.58 ± 1.17^cd^	fat, citrus, green
2-ethylhexanol	104–76-7	1.73 ± 0.03^d^	3.10 ± 0.03^c^	4.46 ± 0.39^b^	9.02 ± 0.37^a^	1.98 ± 0.73^cd^	9.73 ± 0.97^a^	rose, green
benzaldehyde	100–52-7	45.10 ± 1.54^a^	32.97 ± 1.57^b^	15.85 ± 0.49^c^	10.20 ± 0.63^d^	10.51 ± 2.47^d^	2.66 ± 0.47^e^	almond, burnt sugar

Note: Reported values correspond to the mean ± standard deviation. Value in the same row with different letters indicates a significant difference (*p* < 0.05). Flavor descriptions are all from http://www.flavornet.org/flavornet.html.

Polyunsaturated aldehydes, which contribute significantly to off-flavors in food due to their low threshold, are generated from unsaturated fatty acids such as linoleic and linolenic acid through the action of lipoxygenases [42]. In these samples, the relative content of aldehydes was YP (35.76 %), YP-BM (41.04 %), YP-US (46.76 %), YP-US-MP (50.28 %), YP-MP (35.87 %), and YP-MP-US (39.72 %), but the species were reduced to 13, 10, 8, 8, 8, and 7, respectively. Interestingly, butanal (pungent, green), isovaleraldehyde (ethereal, chocolate, peach), and 3-furaldehyde (bread, almond, sweet) were not detected (except for YP-BM) under different treatment conditions as compared to YP material, furthermore, the contents of pentanal (almond, malt, malt), hexanal (grass, tallow, fat), heptanal (fat, citrus, rancid), and benzaldehyde (almond, burnt sugar) in the order of YP > YP-BM > YP-MP > YP-US-MP > YP-US > YP-MP-US. Surprisingly, the concentration of aldehydes and ketones in the YP-MP-US samples was significantly lower, while the level of alcohol was significantly higher compared to the other YP samples. This suggested that synergistic treatment of MP and US induced changes in the composition of volatile flavor substances in yeast proteins, which may be attributed to the conjugating of MP to YP increased the solubility and stability of YP, then the cavitation effect produced and high-energy response environments during the synergistic US to accelerate the reduction of aldehydes or ketones to alcohols. These findings accordingly indicated that the synergistic treatment of MP and US has a pronounced effect on improving the overall flavor of YP.

### 3.3 Characterization of yeast protein structure properties

#### 3.3.1 FTIR analysis

**Fig. 4:**
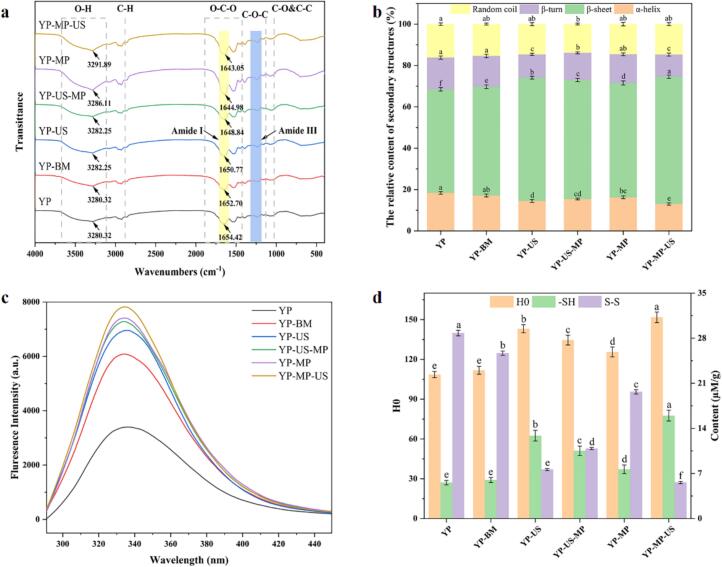
Effect of synergistic treatment of MP and US on YP structural properties. Fourier transform infrared spectroscopy (a) and Relative contents of the secondary structure (b), Intrinsic fluorescence spectroscopy (c), H0, –SH, and S–S content (d).

The polypeptide chain of a protein comprises specific three-dimensional structures, including α-helix, β-sheet, β-turn, and random coil, with alterations in these structures indicating changes in the secondary structure of the protein. Fig. 4a illustrated that synergistic treatment of MP and US significantly affected the secondary structure of YP, leading to interconversion among these structures. Notable changes in the intensities and maximum absorption wavenumbers of specific peaks can be observed, particularly in the O–H stretching region and the amide I band. The absorption peak at nearly 3300 cm^−1^ is caused by the stretching vibration of the amide group N–H and the hydroxyl group –OH [43]. We observed that with the synergistic treatment of MP and US, a red shift occurred in this peak, which may be attributed to local structural rearrangements or the disruption of hydrogen bonds in the YP caused by the combined effect of mannose-binding protein and ultrasound treatment. To analyze the secondary structure of YP samples, we focused on the deconvolution of the amide I band, which primarily reflects the stretching vibration of the C=O group in the protein backbone [44]. Interestingly, the observed red shift in the amide A band suggests that synergistic treatment of MP and US influence the N–H bending vibration of YP. Additionally, slight shifts in the amide I and A bands of MP suggest a possible transition from α-helix to β-sheet structure [45]. In the individual treatments, the relative contents of α-helix and β-turn were reduced, while the relative content of β-sheet was increased in YP-MP samples compared to YP-US. Specifically, a decrease in α-helix content from 17.15 % to 14.46 %, and from 16.31 % to 13.02 %, was observed between YP-BM and YP-US, and between YP-MP and YP-MP-US, respectively. Similarly, the β-turn content decreased from 14.85 % to 11.28 %, and from 13.9 % to 10.7 % (Fig. 4b). These changes suggest that US shifts proteins from an ordered structure to a more flexible and open form, enhancing their functional properties such as emulsification and solubility. The notable increase in β-sheet content indicates that high-intensity US disrupts hydrogen bond stability, converting α-helix structures to β-sheets, which is consistent with the FTIR spectral observations [46]. This finding aligns with the results of Wang et al. [47], who studied the effects of ultrasonic treatment on whey proteins.

In addition, we found that the synergistic treatment of MP and US was effective in improving the degree of denaturation of YP through comparison of YP-US-MP and YP-MP-US samples, which may be attributed to the fact that polysaccharides and proteins work together through the formation of stable complexes or by altering the hydrogen-bonding network. Meanwhile, the synergistic US treatment of YP-MP conjugates resulted in a discernible decrease in α-helix and β-turn content, while the β-sheet content increased significantly within YP (*p* < 0.05). This may be due to the conjugating of MP to YP to form an MP-YP conjugate that makes the YP structure easier to unfold, and the US treatment further accelerates the denaturation of YP.

#### 3.3.2 Intrinsic fluorescence spectroscopy

The intrinsic fluorescence properties of protein tryptophan residues are sensitive to their microenvironment, and changes in them can reflect changes in protein tertiary structure [48]. The fluorescence intensity of the fluorescence spectra for the modified YP tertiary structure in the order of YP-MP-US > YP-MP > YP-US-MP > YP-US > YP-BM > YP (Fig. 4c). We have noticed that the US treatment caused the more intense fluorescence of YP samples through comparison of between YP-BM and YP-US, YP-MP and YP-MP-US, which may be attributed to the fact that US exposes hydrophobic regions of the protein, which places the chromophore in a more hydrophobic environment, resulting in enhanced fluorescence intensity [49]. Interestingly, we found that the fluorescence intensity of YP-MP conjugates (YP-MP-US), was significantly higher than that of YP samples without MP conjugating pretreatment (YP-US-MP) (*p* < 0.05). This could be attributed to the formation of conjugates from polysaccharides and proteins, which were exposed to more YP hydrophobic groups through US treatment. Similarly, research pointed out that the YP-MP conjugates pre-treatment resulted in protein structure unfolding and being more susceptible to ultrasound [50].

#### 3.3.3 Surface hydrophobicity (H0)

Surface hydrophobicity is a crucial indicator for evaluating changes in protein denaturation and physicochemical properties. As illustrated in Fig. 4d, the highest H0 level (151.75 ± 3.99) was observed for the YP-MP-US. Among them, the H0 levels were YP (108.56 ± 2.33), YP-US (143.09 ± 2.99), and YP-MP (125.60 ± 3.66), which indicated US and MP treatment alone led to significantly higher levels of H0. Meanwhile, the H0 levels of YP-US and YP-MP-US were significantly increased compared to the YP-BM (111.78 ± 2.99) and YP-MP sample, this further suggested that US treatment has a significant effect on increasing H0 levels of YP.

In addition, the H0 level of YP-MP-US was significantly higher than that of YP-US-MP (134.54 ± 3.66), which could be attributed to the addition of MP to form the YP-MP conjugates, the structure of YP partially unfolded under cointegrate treatment of MP, and subsequent synergistic US treatment caused cavitation and thermal effects that cleaved disulfide bonds, exposing more internal hydrophobic groups on the protein surface and significantly raising the H0 level [51,52]. Lee et al. [53] noted that US treatment can expose hydrophobic groups previously buried within protein molecules, enhancing their emulsifying properties. Similarly, Yin et al. [54] found that proteins with higher hydrophobicity exhibit higher emulsifying values. In this study, the YP-MP-US sample showed the highest hydrophobicity and EAI (64.76 m^2^/g, Fig. 1c). Moreover, a hydrophobic environment promotes the formation of β-sheet structures, as the exposure of hydrophobic residues prevents water from filling the gaps between unfolded molecules, thereby favoring β-sheet formation [55]. Consequently, the high hydrophobicity of the YP-MP-US sample is associated with increased β-sheet content.

#### 3.3.4 Sulfhydryl group (–SH) and disulfide bond (S–S) contents

The content of –SH groups and S**–**S bonds plays a crucial role in protein structure and function, especially playing critical roles in protein folding, stability, and functional regulation [56]. As shown in Fig. 4d, the YP-MP-US sample exhibited the highest –SH groups content (15.97 ± 0.83 μM/g) and the lowest S–S contents (5.59 ± 0.17 μM/g). Notably, there was a significant difference between YP-BM and YP-US, YP-MP and YP-MP-US samples. The ultrasonically treated samples exhibited a significant increase in –SH groups and a significant decrease in S–S bonds. This phenomenon is attributed to the high-intensity ultrasound forces generated during ultrasonic cavitation, which cleave S–S bonds, thereby increasing the formation of –SH groups with prolonged US treatment. Furthermore, prolonged ultrasonic exposure has been proven to promote the decomposition of water molecules into reactive free radicals, such as H• and HO• [57]. Furthermore, the –SH group content was significantly higher in YP-MP-US samples, and S–S content was significantly lower compared to YP-US-MP, this phenomenon was probably on account of MP treatment results in the clustering of YP-MP conjugates, which prevented the protein from fully unfolding and thereby decreased the free –SH content [58]. It is consistent with the results of Wang et al. [59] on US treatment of soybean isolate protein complexes with different concentrations of inulin. Previous studies have demonstrated that improving the particle size of proteins would expose –SH groups on the surface and disrupt the S–S bonds that stabilize protein aggregates, which is consistent with the results of the present study [60].

#### 3.3.5 SEM

**Fig. 5:**
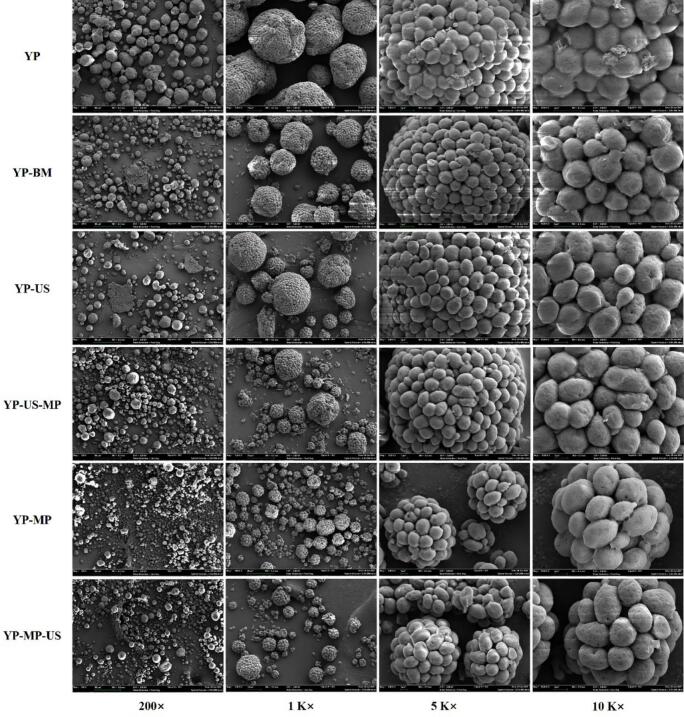
Effect of synergistic treatment of MP and US on SEM of YP samples.

The synergistic effects of MP and US treatment on micro-conformation the of YP are shown in Fig. 5. YP exhibits a relatively irregular spherical shape with many irregular protein cluster structures, while the agglomerated state of the YP-MP-US sample was significantly smaller than that of the other YPs (*p* < 0.05), suggested that the particle size and aggregation of the YP-MP-US sample were significantly improved. Similarly, we found that the ultrasonicated YP samples showed a looser and less agglomerated through comparison of between YP-BM and YP-US, YP-MP and YP-MP-US, suggesting that proper US treatment can significantly improve the microstructure of YP. The US treatment likely unfolded protein molecules and increased the exposure of –SH and hydrophobic groups, which facilitated their interactions and helped to prevent untreated protein aggregates [61,62]. In addition, we noticed that the synergistic MP and US treatment on YP samples (YP-MP-US) presented a looser aggregation state than that of YP samples without MP conjugating pretreatment (YP-US-MP), which may be due to the ultrasound-induced cavitation effect during sonication and the local temperature increase due to mechanical shear forces that tightly binds the proteins and polysaccharides, which enhances covalent attachment to polysaccharides [63]. These samples are thought to easily form a reticulated structure at the air–water interface, which traps more water molecules, forms a thicker interfacial film consisting of conjugates, and retains more water, thereby improving foam stability [64].

## 4 Conclusions

In this study, the synergistic treatment of MP and US effectively increased the soluble content of YP and improved its overall flavor. The integrated approach effectively reduced the particle size of soluble YP and improved its solubility, H0, foaming properties, free –SH groups, and ζ-potential. The synergistic MP and US treatment exposed protein hydrophobic groups and yielded the highest H0 values (151.75 ± 3.99), particularly in the samples of YP-MP-US. FTIR measurements revealed an increase in β-sheets alongside a decrease in α-helix and β-turns, while intrinsic fluorescence spectroscopy indicated enhanced fluorescence intensity. Similarly, the results of sensory evaluation, E-tongue, E-nose, and HS-SPME-GC–MS demonstrated that the synergistic treatment significantly improved the characteristic yeast flavor of YP (*p* < 0.05). Overall, protein modification with the synergistic treatment of MP and US proved highly effective, especially for YP with substantial insoluble components. Thus, modified YP shows potentiality as a supplement to traditional proteins in the food industry, laying the foundation for further research and exploration of other protein modification methods in the future.

## CRediT authorship contribution statement

**Jin Luo:** Writing – original draft, Methodology, Investigation, Data curation. **Li Liang:** Writing – original draft, Formal analysis, Conceptualization. **Yongzhao Bi:** Software. **Xialei Liu:** Data curation. **Kaina Qiao:** Software. **Zunying Liu:** Supervision. **Xiangzhao Mao:** Conceptualization. **Baoguo Sun:** Supervision, Resources. **Yuyu Zhang:** Writing – review & editing, Funding acquisition, Conceptualization.

## Declaration of competing interest

The authors declare that they have no known competing financial interests or personal relationships that could have appeared to influence the work reported in this paper.
